# The association between experience of COVID-19-related discrimination and psychological distress among healthcare workers for six national medical research centers in Japan

**DOI:** 10.1007/s00127-023-02460-w

**Published:** 2023-03-17

**Authors:** Rachana Manandhar Shrestha, Yosuke Inoue, Shohei Yamamoto, Ami Fukunaga, Makiko Sampei, Ryo Okubo, Naho Morisaki, Norio Ohmagari, Takanori Funaki, Kazue Ishizuka, Koushi Yamaguchi, Yohei Sasaki, Kazuyoshi Takeda, Takeshi Miyama, Masayo Kojima, Takeshi Nakagawa, Kunihiro Nishimura, Soshiro Ogata, Jun Umezawa, Shiori Tanaka, Manami Inoue, Maki Konishi, Kengo Miyo, Tetsuya Mizoue

**Affiliations:** 1 Department of Epidemiology and Prevention, Center for Clinical Sciences, National Center for Global Health and Medicine, 1-21-1 Toyama, Shinjuku-Ku, Tokyo, 162-8655 Japan; 2 Department of Health Science, Health Promotion, Nippon Sport Science University, Tokyo, Japan; 3 Clinical Research and Education Promotion Division, National Center Hospital, National Center of Neurology and Psychiatry, Tokyo, Japan; 4 Department of Social Science, National Research Institute for Child Health and Development, Tokyo, Japan; 5 Disease Control and Prevention Center, National Center for Global Health and Medicine, Tokyo, Japan; 6 Division of Infectious Diseases, Department of Medical Subspecialties, National Center for Child Health and Development, Tokyo, Japan; 7 Center of Maternal-Fetal, Neonatal and Reproductive Medicine, National Center for Child Health and Development, Tokyo, Japan; 8 National Center Hospital, National Center of Neurology and Psychiatry, Tokyo, Japan; 9 Department of Frailty Research, Research Institute, National Center for Geriatrics and Gerontology, Aichi, Japan; 10 Department of Social Science, Research Institute, National Center for Geriatrics and Gerontology, Aichi, Japan; 11 Department of Preventive Medicine and Epidemiology, National Cerebral and Cardiovascular Center, Osaka, Japan; 12 Division of Cohort Research, Institute for Cancer Control, National Cancer Center, Tokyo, Japan; 13 Division of Prevention, Institute for Cancer Control, National Cancer Center, Tokyo, Japan; 14 Center for Medical Informatics Intelligence, National Center for Global Health and Medicine, Tokyo, Japan

**Keywords:** COVID-19, Healthcare workers, Discrimination, Psychological distress, Mental health

## Abstract

**Background:**

Discrimination is an important determinant of negative mental health outcomes. This study determined the association between the experience of COVID-19-related discrimination and psychological distress among healthcare workers (HCWs) in Japan.

**Methods:**

This cross-sectional study conducted a health survey among 5703 HCWs of six national medical and research centers in Japan from October 2020 to March 2021. COVID-19-related discrimination was defined either when participants or their family members were badmouthed or when they felt discriminated against in some way. We used the Kessler Psychological Distress Scale (K6) to assess the presence of severe psychological distress (≥ 13 points). We used logistic regression models to examine the association between discrimination and psychological distress. We also identified factors associated with discrimination.

**Results:**

Of the participants, 484 (8.4%) reported COVID-19-related discrimination and 486 (8.5%) had severe psychological distress. HCWs who were female vs. male (adjusted odds ratio [AOR] = 1.41, 95% confidence interval [CI] = 1.28–1.55), had high vs. low viral exposure (AOR = 2.31, 95% CI = 1.81–2.93), and worked for 11 or more hours/day vs. 8 or less hours/day (AOR = 1.42, 95% CI = 1.35–1.49) were more likely to have experienced COVID-19-related discrimination. The AOR (95% CI) of severe psychological distress was 1.83 (1.29–2.59) among those who experienced discrimination. In the stratified analysis by sociodemographic and job-related factors, all the interactions did not reach statistical significance (p for interaction > 0.20).

**Conclusion:**

Experience of COVID-19-related discrimination was associated with severe psychological distress among HCWs. During the pandemic, effective measures should be taken to prevent the development of negative mental health outcomes in HCWs who experience discrimination.

## Introduction

Since the emergence of ongoing coronavirus disease 2019 (COVID-19) pandemic, it has become a global health threat. Healthcare workers (HCWs), particularly those involved in COVID-19-related patient care were at a heightened risk of infection [1, 2]. For example, a meta-analysis, including 28 studies from seven countries, reported that the percentage of HCWs who tested positive for COVID-19 was as high as 51.7% [1]. A prospective cohort study among community individuals and frontline HCWs reported that compared to the community individuals, frontline HCWs had 12-fold higher risk of reporting infection [2].

Since the beginning of the COVID-19 pandemic, the fear of transmission of the severe acute respiratory syndrome coronavirus 2 (SARS-CoV-2) from HCWs to the general population [3] provoked rapid stigma and discrimination towards HCWs, particularly against those involved in care of COVID-19 patients [4–6]. It was reported that HCWs faced discrimination in the form of verbal attacks and threats [3], avoidance from family and community members [7], avoidance from community members towards their family [8], and stigmatization [9]. Although fewer numbers of infections and death from COVID-19 have been reported in Japan compared to many other countries [10], a few studies reported that frontline HCWs and their family members have experienced discrimination [11, 12]. For instance, children of HCWs were refused access to kindergartens, school, and childcare facilities [11, 12].

Discrimination is an important determinant of negative mental health outcomes [13]. Pathways that can link discrimination to mental health include the direct effect of discrimination, psychological stress response to decreased positive emotion and increased negative emotion, and the deterioration of health-related behaviors [13]. Given the concern regarding stigma and discrimination associated with COVID-19 during current pandemic, such experiences can lead to negative mental health consequences among the HCWs. For example, previous studies from the Philippines and Spain reported that those who perceived a higher level of discrimination during the current pandemic had poor mental health [14], and depression symptoms, psychological distress and death thoughts [15]. Discrimination experience was also associated with higher professional-turnover intention [14], which can eventually affect their work outcomes. A survey in Japan among 4386 HCWs reported that 19.1% felt avoided by their family members and friends [16]. However, there has been no study on the association between COVID-19-related discrimination and mental health among Japanese HCWs.

Thus, this study explored the factors associated with COVID-19-related discrimination and examined the association between experience of COVID-19-related discrimination and psychological distress among the staff of national medical research centers in Japan. We hypothesized that the experience of COVID-19-related discrimination could be positively associated with psychological distress among the HCWs. Furthermore, given that a certain group of HCWs (e.g., females and frontline workers) might be more susceptible to stigma and discrimination than other groups, we also hypothesized that the magnitude of association between discrimination and psychological distress may differ across subgroups in relation to socio-demographic and job-related factors.

## Methods

### Study design and participants

A multi-center collaborative study has been conducted among the staff members (mostly HCWs) of the six National Centers for Advanced Medical and Research in Japan to monitor the transmission of SARS-CoV-2. Each national center conducted a serological test and questionnaire survey at least once per year during the COVID-19 epidemic since 2020. Written informed consent was obtained from all the participants. After completing the opt-out process, the survey data were anonymized and submitted to the study committee for pooled analysis. The study design and procedure for data collection at each center were approved by the ethical committee of each center, while those of pooling study were approved by that of the National Center for Global Health and Medicine (NCGM) (approval number: NCGM-G-004233). For the current study, we used the data collected from the surveys conducted between October 2020 and March 2021 before the vaccination program at each center [17].

**Fig. 1:**
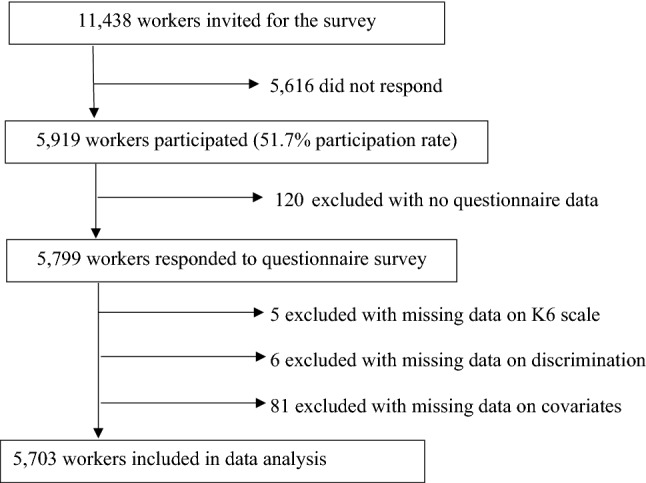
Flow diagram of study participants

Of the 11,438 staff members invited for the survey, 5919 participated (51.7% participation rate) (Fig. 1). We requested all the eligible participants to complete a questionnaire survey. After excluding participants without questionnaire data (n = 120), with missing information on exposure (n = 6), outcome (n = 5), and selected covariates (described below) (n = 81), 5703 participants were included for the statistical analysis.

### Measures

#### Psychological distress assessment (K6 scale)

The Japanese version of the Kessler Screening Scale for Psychological Distress (K6) scale was used to assess psychological distress [18]. It included six questions that rated participants’ frequency of how often they felt (1) nervous, (2) hopeless, (3) restless or fidgety, (4) so depressed that nothing could cheer them up, (5) that everything was an effort, and (6) worthless during the past 30 days. The responses options included five options, which ranged from “always” (score = 4) to “not at all” (score = 0) and the total score ranged from 0 to 24. Participants were judged to have severe psychological stress if the score was ≥ 13 points [19].

#### Discrimination

We asked the participants if they had the following two experiences in relation to COVID-19 pandemic: “I or my family have been bad-mouthed” (yes or no) and “I felt that I was discriminated against in some way” (yes or no). We did not specify the time frame for discrimination experience and the timing of such experience, if any. If participants answered “yes” to at least one of the questions, they were considered to have experienced COVID-19-related discrimination. These questions were developed specifically for this survey based on a previous study conducted on COVID-19-related stigma among HCWs in Vietnam [20].

#### Covariates

We obtained participant’s information on the following covariates: sex (male or female), age, living arrangement, job category, COVID-19-related works, working hours, smoking status, alcohol consumption, physical activity, sleep duration, height, weight, and comorbidity.

Job category included doctors, nurses, allied healthcare professionals, researchers, administrative and management staffs, and we merged researchers, administrative and management staffs into the category “non-clinical staffs”. Regarding degree of occupational exposure to SARS-CoV-2, we initially asked participants about their engagement in COVID-19-related work, and asked the following two questions: “Have you ever engaged in the COVID-19 related work?” (yes or no) and “Did you engage in any work in which you were heavily exposed to the SARS-CoV-2?” (yes or no). We then defined the degree of occupational exposure to SARS-CoV-2 at work and categorized the participants into three groups according to potential risk of infection: low (not engaged in COVID-19 related work), moderate (engaged in COVID-19 related work without heavy exposure to the virus), and high (engaged in COVD-19 related work with heavy exposure to the virus).

Smoking status was categorized into three groups (never, former, or current smoker) based on participants’ responses on smoking conventional cigarettes and use of heated tobacco products (IQOS, glo, PULZE, WEECKE, etc.). Alcohol consumption was estimated based on the information on the consumption frequency and amount consumed in a day in “*go*” (*go* is a Japanese traditional unit equivalent to about 180 ml). Leisure-time physical activity was measured in minute/week, based on one question about the weekly time spent on either indoor or outdoor physical activity.

Height and weight were used to calculate body mass index (BMI). We defined co-morbid condition if they had any one of the following chronic conditions: diabetes, hypertension, chronic obstructive pulmonary disease (COPD), heart disease, cerebrovascular disease, cancer, and other chronic diseases.

### Statistical analysis

We conducted logistic regression analysis to investigate COVID-19-related discrimination in relation to age (< 30, 30 to  < 40, 40 to  < 50, or ≥ 50 years), sex (male or female), living arrangement (living alone or living with others), job category (doctor, nurse, allied healthcare professional, or non-clinical staffs), degree of occupational exposure to SARS-CoV-2 (low, moderate, or high), and working hours (≤ 8, 9–10, or ≥ 11 h/day). Then, we examined the association between discrimination and severe psychological distress using logistic regression. In model 1, we adjusted for age, sex, and living arrangement. In model 2, we additionally adjusted for job category, degree of occupational exposure to SARS-CoV-2, working hours, comorbidity (yes or no), and BMI (< 18.5, 18.5 to  < 23, 23 to  < 25, 25 to < 30, or ≥ 30 kg/m^2^). In model 3, we further adjusted for smoking status (never, former, or current smoker), alcohol consumption (none, < 1, 1 to  < 2, or ≥ 2 *go*/day), sleep duration (< 6, 6 to  < 7, or ≥ 7 h), and leisure-time physical activity (none, < 1 h/week, 1 to  < 2 h/week, or ≥ 2 h/ week) as possible mediators. We conducted stratified analyses by socio-demographic (age, sex, and living arrangement) and occupation-related factors (job categories, degree of occupational exposure to SARS-CoV-2, and working hours) to examine if the associations between discrimination and psychological distress differ across the groups and tested p for interactions. We reported odds ratio (OR) and 95% confidence interval (CI) for logistic regression and the level of significance was set at p < 0.05 (two-tailed). We used Stata version 15 (College Station, TX, USA) for all statistical analyses.

## Results

Table 1 presents the characteristics of study participants. Among 5703 participants, 484 (8.4%) participants reported that they experienced a COVID-19-related discrimination (being bad-mouthed and/or experienced some sort COVID-19-related discrimination). In this study, 23.7% of the participants were below 30 years old, 70.2% were female and 67.5% were living with others. Regarding occupational background, 33.2% were nurses and more than half of the participants (59.7%) had a lower degree of occupational exposure to SARS-CoV-2.

**Table 1:** Characteristics of study participants of the six national medical research centers in Japan

		Discrimination	
		No	Yes
Total	5703	5218 (100)	484 (100)
Age			
< 30 years	1353	1221 (23.4)	132 (27.3)
30– < 40 years	1464	1328 (25.5)	136 (28.1)
40– < 50 years	1568	1441 (27.6)	127 (26.2)
≥ 50 years	1318	1229 (23.6)	89 (18.4)
Sex			
Male	1702	1591 (30.5)	111 (22.9)
Female	4001	3628 (69.5)	373 (77.1)
Living arrangement			
Living alone	1851	1661 (31.8)	190 (39.3)
Living with others	3852	3558 (68.2)	294 (60.7)
Job category			
Doctors	806	745 (14.3)	61 (12.6)
Nurses	1895	1664 (31.9)	231 (47.7)
Allied healthcare professionals	998	922 (17.7)	76 (15.7)
Non-clinical staffs	2004	1088 (36.2)	63 (24.0)
Degree of occupational exposure to SARS-CoV-2			
Low	3407	3197 (61.3)	210 (43.4)
Moderate	1181	1068 (20.4)	113 (23.4)
High	1115	954 (18.3)	161 (33.3)
Working hours			
≤ 8 h /day	3027	2811 (53.9)	216 (44.6)
9–10 h/day	1951	1758 (33.7)	193 (39.9)
≥ 11 h/day	725	650 (12.4)	75 (15.5)
Body mass index			
< 18.5 kg/m^2^	631	577 (11.0)	54 (11.3)
18.5– < 23 kg/m^2^	3387	3093 (59.3)	294 (60.7)
23– < 25 kg/m^2^	832	767 (14.7)	65 (13.4)
25– < 30 kg/m^2^	697	643 (12.3)	54 (11.1)
≥ 30 kg/m^2^	156	139 (2.7)	17 (3.5)
Comorbidity			
No	4350	4008 (76.8)	342 (70.7)
Yes	1353	1211 (23.2)	142 (29.3)
Smoking status			
Never	4669	4280 (82.0)	389 (80.4)
Former	678	625 (12.0)	53 (10.9)
Current	356	314 (6.0)	42 (8.7)
Alcohol consumption			
None	2155	1976 (37.8)	179 (37.0)
< 1 *go*/day	2931	2682 (51.4)	249 (51.4)
1– < 2 *go*/day	487	442 (8.5)	45 (9.3)
≥ 2 *go*/day	130	119 (2.3)	11 (2.3)
Leisure-time physical activity			
None	1436	1323 (25.4)	113 (23.4)
< 1 h/week	2349	2165 (41.5)	184 (38.0)
1– < 2 h/week	955	864 (16.5)	91 (18.8)
≥ 2 h/week	963	867 (16.6)	96 (19.8)
Sleep duration			
< 6 h/day	2692	2442 (46.8)	250 (51.6)
6– < 7 h/day	2052	1892 (36.2)	160 (33.1)
≥ 7 h/day	959	885 (17.0)	74 (15.3)

Figures in the table are number (%)

Table 2 shows the results of multiple logistic regression analysis investigating the socio-demographic and job-related factors associated with COVID-19-related discrimination. In bivariable analysis, female staff (COR = 1.47, 95% CI 1.26–1.71), nurses (COR = 2.26, 95% CI 1.32–3.36), those having moderate (COR = 1.61, 95% CI 1.43–1.81) and higher degree of occupational exposure to SARS-CoV-2 (COR = 2.56, 95% CI 1.95–3.38), and those working 9–10 h/day (COR = 1.42, 95% CI 1.21–1.68) and 11 or more hours/day (COR = 1.50, 95% CI 1.32–1.70) were more likely to have the experience of COVID-19-related discrimination compared to their counterparts, whereas those living with others were less likely to have the experience compared with those living alone (COR = 0.72, 95% CI 0.61–0.85). In multivariable analysis including all covariates, the associations remained significant for sex, degree of occupational exposure to SARS-CoV-2, and working hours, but the associations for occupation and living arrangement were attenuated and became statistically not significant.

**Table 2:** Factors associated with COVID-19-related discrimination among study participants of the six national medical research centers in Japan

	Discrimination n (%)	COR (95% CI)	AOR (95% CI)^a^
Age			
< 30 years	132 (9.8)	Reference	Reference
30– < 40 years	136 (9.3)	0.94 (0.65–1.36)	1.24 (0.91–1.69)
40– < 50 years	127 (8.1)	0.81 (0.60–1.10)	1.13 (0.87–1.46)
≥ 50 years	89 (6.8)	0.66 (0.42–1.06)	1.11 (0.81–1.54)
Sex			
Male	111 (6.5)	Reference	Reference
Female	373 (9.3)	1.47 (1.26–1.71)	1.41 (1.28–1.55)
Living arrangement			
Living alone	190 (10.3)	Reference	Reference
Living with others	294 (7.6)	0.72 (0.61–0.85)	0.90 (0.74–1.10)
Job category			
Non-clinical staffs	61 (7.6)	Reference	Reference
Doctors	231 (12.2)	1.33 (0.86–2.04)	0.83 (0.53–1.30)
Nurses	76 (7.6)	2.26 (1.32–3.36)	1.43 (0.87–2.36)
Allied healthcare professionals	63 (5.8)	1.34 (0.93–1.92)	1.14 (0.79–1.67)
Degree of occupational exposure to SARS-CoV-2			
Low	210 (6.2)	Reference	Reference
Moderate	113 (9.6)	1.61 (1.43–1.81)	1.50 (1.27–1.78)
High	161 (14.4)	2.56 (1.95–3.38)	2.31 (1.81–2.93)
Working hours			
≤ 8 h/day	216 (7.1)	Reference	Reference
9–10 h/day	193 (9.9)	1.42 (1.21–1.68)	1.26 (1.12–1.41)
≥ 11 h/day	75 (10.3)	1.50 (1.32–1.70)	1.42 (1.35–1.49)

*CI* confidence interval, *COR* crude odds ratio, *AOR* adjusted odds ratio

^a^Model was adjusted for age (< 30, 30- < 40, 40- < 50, or ≥ 50 years), sex (male or female), living arrangement (living alone or living with others), job category (doctors, nurses, allied health care professionals, or non-clinical staffs), degree of occupational exposure to SARS-CoV-2 (low, moderate, or high), and working hours (≤ 8, 9–10, or ≥ 11 h/day)

Table 3 shows the association between COVID-19-related discrimination and psychological distress. A total of 486 (8.5%) had severe psychological distress. Compared with participants without experience of discrimination, those who had such experience had a significantly higher odds of having psychological distress in model 2 (AOR = 1.79, 95% CI 1.26–2.56). Further adjustment of health behaviors (model 3) did not materially change the estimate) (AOR = 1.83, 95% CI 1.29–2.59). In stratified analyses, the association appears stronger among clinical staffs: doctors (AOR = 1.99, 95% CI 1.15–3.46), nurses (AOR = 1.83, 95% CI 1.25–2.68), and allied healthcare workers (AOR = 2.49, 95% CI 1.62–3.84), compared with non-clinical staffs (AOR = 1.38, 95% CI 0.70–2.68). The association appears stronger among HCWs with high degree of occupational exposure to the virus (AOR = 2.58, 95% CI 1.59–4.18) than those with low degree of the exposure (AOR = 1.80, 95% CI 0.95–3.41). However, all the interactions did not reach statistical significance (p for interaction > 0.20).

**Table 3:** Association between discrimination and severe psychological distress among the total and subgroups of study population

	Psychological distress, n (%)	AOR (95% CI) associated with discrimination experience	P for interaction^a^
Total participants			
Model 1	486 (8.5)	1.96 (1.35–2.82)	–
Model 2	486 (8.5)	1.79 (1.26–2.56)	–
Model 3	486 (8.5)	1.83 (1.29–2.59)	**–**
Subgroup of participants^b^			
Age			0.25
< 30 years	141 (10.4)	1.45 (0.67–3.10)	
30– < 40 years	145 (9.9)	2.10 (1.74–2.53)	
40– < 50 years	128 (8.2)	2.15 (1.43–3.23)	
≥ 50 years	72 (5.5)	1.91 (0.90–4.06)	
Sex			0.78
Male	145 (8.5)	1.72 (0.78–3.77)	
Female	341 (8.5)	1.89 (1.45–2.46)	
Living arrangement			0.83
Living alone	200 (10.8)	1.68 (1.09–2.61)	
Living with others	286 (7.4)	1.91 (1.20–3.05)	
Job category			0.28
Doctors	49 (6.1)	1.99 (1.15–3.46)	
Nurses	201 (10.6)	1.83 (1.25–2.68)	
Allied healthcare professionals	81 (8.1)	2.49 (1.62–3.84)	
Non-clinical staffs	77 (9.0)	1.38 (0.70–2.68)	
Degree of occupational exposure to SARS-CoV-2			0.24
Low	265 (7.8)	1.80 (0.95–3.41)	
Moderate	93 (7.8)	1.13 (0.61–2.10)	
High	128 (11.5)	2.58 (1.59–4.18)	
Working hours			0.68
≤ 8 h/day	220 (7.3)	2.02 (1.52–2.68)	
9–10 h/day	175 (9.0)	1.72 (1.18–2.50)	
≥ 11 h/day	91 (12.6)	2.10 (0.57–7.73)	

Model 1 was adjusted for age (< 30, 30 to < 40, 40 to < 50, or ≥ 50 years), sex (male or female), and living arrangement (living alone or living with others). Model 2 was additionally adjusted for job category (doctors, nurses, allied health care professionals, or non-clinical staffs), degree of occupational exposure to SARS-CoV-2 (low, moderate, or high), working hours (≤ 8, 9–10, or ≥ 11 h/day), comorbidity (yes or no), and BMI (< 18.5, 18.5 to < 23, 23 to < 25, 25 to < 30, or ≥ 30 kg/m^2^). Model 3 was additionally adjusted for smoking (never, former, current), alcohol consumption (none, < 1, 1 to < 2, or ≥ 2 *go*/day), sleep duration (< 6, 6 to  < 7, or ≥ 7 h), and leisure-time physical activity (none, < 1, 1 to < 2, or ≥ 2 h/week)

*CI* confidence interval, *AOR* adjusted odds ratio

^a^Derived from the joint test for the interaction between discrimination and covariates using “Contrast” command in Stata

^b^Adjusted for all the covariates in model 3

## Discussion

In this study, we identified female sex, the degree of occupational exposure to SARS-CoV-2, and working hours as factors associated with COVID-19-related discrimination. Furthermore, the experience of COVID-19-related discrimination was positively associated with psychological distress. When we conducted stratified analysis by socio-demographic and occupational factors, all the interactions did not reach statistical significance.

We found that female staff were more likely to have experience of COVID-19-related discrimination compared with male staff. This finding was consistent with a previous study by Elhadi et al. [21] that reported higher stigmatization among female HCWs compared to male counterparts (36.1% vs. 28.2%). Staffs with higher exposure to SARS-CoV-2 being discriminated more is also comparable to a previous study by Yadav et al. [22] that showed higher perceived stigma among those working in high risk areas than those in low risk areas (73.7% vs. 67.4%). Furthermore, in this study, higher proportions of nurses (12.2%) experienced discrimination, followed by doctors and allied healthcare professions (7.6% each) and non-clinical staffs (5.6%). This finding is in line with that of a previous study by Zandifar et al. [23] that reported higher discrimination among physicians and nurses than technicians. Regarding working hours, we found that those working longer hours tended to perceive higher discrimination. Healthcare workers with long working hours are at higher risk for burnout [24], which could cause emotional exhaustion and have negative feelings about work [25] and might perceive higher sense of discrimination.

Increased psychological distress associated with COVID-19-related discrimination observed in this study agrees with that of a meta-analysis by Schubert et al., which reported a positive association of stigmatization from work-related COVID-19 exposure with depression and anxiety among HCWs during the COVID-19 pandemic [26]. These findings suggest that COVID-19-related discrimination could be harmful to mental health and should be addressed to ensure better mental health among frontline HCWs.

Health behaviors can influence mental health and the deterioration of health-related behaviors induced by discrimination experience has been proposed as one of the pathways linking discrimination to psychological distress [13]. In the present study, however, the proportion of participants with and without discrimination experience was similar according to health behaviors and that the association between COVID-19-related discrimination and psychological distress was virtually unchanged after adjusting for health behaviors. We may therefore conclude that the observed association between discrimination experience and psychological distress may not be ascribed to deteriorated health behaviors.

Discrimination towards HCWs might have changed during the pandemic. As the present study was conducted during the early phase of the pandemic before vaccine rollout, HCWs might have greater chance of suffering frequent and severe COVID-19-related discrimination from others due to the fear of infection. In fact, a study among the general population in the US [27] reported that experiences of discrimination peaked early in the pandemic. In the present study, we did not ask the timing of the discrimination experiences. Most recent experience of discrimination might have greater impact on the mental health at the time of this survey. It is also possible that discrimination experienced during the early period (probably more frequent and severer than recent ones) might have a stronger and long-lasting impact on psychological distress. Additional study is required to examine the differential impact of discrimination in relation to the timing of such experience.

The strength of associations between COVID-19-related discrimination and psychological distress may differ according to socio-demographic (age, sex and living arrangement) and job-related factors (job categories, degree of occupational exposure to SARS-CoV-2 and working hours). Contrary to the expectation, we did not find any strong evidence of statistically significant interactions while the point estimates of the associations were higher among female staff, clinical staffs (doctors, nurses, and allied healthcare workers), HCWs with high risk of occupational exposure to the virus. A possible explanation for these findings could be that the clinical staff and those who had higher degree of occupational exposure to virus were the ones involved in the treatment of COVID-19 patients. This could have made them more vulnerable to psychological distress associated with COVID-19-related discrimination they faced. Higher estimates of psychological distress among female staff in this study could be since they were more sensitive to COVID-19-related discrimination because of pre-existing discrimination and inequality against females [28].

The major strength of the present study includes large number of participants from six different national medical research centers in Japan. However, some limitations should be acknowledged. First, the information used in this study was self-reported, which could be subject to recall bias. Second, as the questionnaire included sensitive questions on mental health issues, responses could have been subject to social desirability bias. Third, we did not ask about the frequency, timing, and intensity of discrimination experience. Therefore, we cannot assess the psychological impact of discrimination experience from these aspects. Fourth, we assessed psychological distress using a self-administered questionnaire via the K6 scale without administration by a psychiatrist. However, the scale has been validated in Japan [18]. Fifth, the questions for assessment of discrimination have not been validated. Sixth, because of the cross-sectional data, we do not know whether the associations are causal. Lastly, this study was conducted among those working in the healthcare and research centers, thus the findings may not be generalizable for other settings.

## Conclusion

This study provided evidence on the association between the experience of COVID-19-related discrimination and psychological distress among the HCWs from the six national healthcare centers in Japan. Our findings highlight the need of support for those who have suffered from mental health problems due to COVID-19-related discrimination.

## Data Availability

The data underlying this article cannot be shared publicly due to ethical restrictions and participant confidentiality concerns, but de-identified data are available from Dr. Mizoue (Department of Epidemiology and Prevention, Center for Clinical Sciences, National Center for Global Health and Medicine, Tokyo, Japan) to qualified researchers on reasonable request.
